# Divergent contributions of autistic traits to social psychological knowledge

**DOI:** 10.1073/pnas.1915787116

**Published:** 2019-12-10

**Authors:** Emily C. Taylor, Lucy A. Livingston, Mitchell J. Callan, Punit Shah

**Affiliations:** ^a^Department of Psychology, University of Bath, Bath BA2 7AY, United Kingdom;; ^b^Social, Genetic and Developmental Psychiatry Centre, Institute of Psychiatry, Psychology and Neuroscience, King’s College London, London SE5 8AF, United Kingdom;; ^c^School of Psychology, Cardiff University, Cardiff CF10 3AT, United Kingdom

We examined Gollwitzer et al.’s (1) study on autism and better social psychological knowledge with interest, given its large samples and open dataset. We commend the authors for raising the bar for autism research, which is typically underpowered and rarely draws on social psychology. Notably, their research follows recent investigations of autism-related strengths, and findings that certain social processes (e.g., social motivation) are unimpaired in autistic people who compensate for their social difficulties (2–4). Gollwitzer et al.’s article, together with recent research, is a move toward greater appreciation for autism and neurodiversity in society (5).

Notwithstanding our enthusiasm for the research, we are surprised that a positive relationship between an autism trait questionnaire and social knowledge is found (1). As the autism questionnaire is a clinical screening tool (6), it measures a wide range of traits (e.g., social understanding, attention to detail), reflecting the fractionation of diagnosable autism (7). It does not have an optimal factor structure to measure autism as a unitary construct, as confirmed by reanalyzing Gollwitzer et al.’s data. Unifactorial model fit indices—root mean square error of approximation = 0.10 and Tucker−Lewis Index = 0.62—are suggestive of poor fit, as they are >0.08 and <0.90, respectively. Therefore, given the absence of any subscales (as reported by the authors), we are left wondering whether any of the questionnaire’s items—that is, different autistic traits—are individually correlated with social psychological knowledge. This powerful analytic approach, used in large-scale studies (8), is possible, given the large dataset.

Following previous theory (9), we suspect that different autistic traits have divergent associations with social psychological knowledge. Indeed, some traits are linked to greater social knowledge in line with Gollwitzer et al. (1); however, others are associated with lower scores (Table 1). Broadly speaking, nonsocial traits are linked to better performance, whereas social traits are negatively related to social psychological knowledge. For example, the correlation between social knowledge and responses to “If there is an interruption, I can switch back to what I was doing very quickly” is in completely the opposite direction from the correlation with “When I’m reading a story, I find it difficult to work out the characters’ intentions” (*z* = 24.13, *P* < 0.001). This highlights the striking extent to which different autistic traits have divergent relationships with social psychological knowledge, and most correlations are significantly different from the link between autism and social knowledge reported by the authors (see Table 1).

**Table 1.** Links between individual autistic traits and social psychological knowledge

Autistic trait questionnaire (6) item	*r*	*z*	*β*
I often notice small sounds when others do not	0.02	−1.89	0.03*
I usually concentrate more on the whole picture, rather than the small details (R)	0.09***	2.84**	0.06***
I find it easy to do more than one thing at once (R)	0.17***	10.07***	0.11***
If there is an interruption, I can switch back to what I was doing very quickly (R)	0.20***	12.65***	0.15***
I find it easy to ‘read between the lines’ when someone is talking to me (R)	0.01	−3.55***	−0.03
I know how to tell if someone listening to me is getting bored (R)	0.03*	−1.61	0.02
When I’m reading a story, I find it difficult to work out the characters’ intentions	−0.19***	−18.51***	−0.18***
I like to collect information about categories of things (e.g., types of car, types of bird etc.)	−0.03*	−5.66***	−0.02
I find it easy to work out what someone is thinking or feeling just by looking at their face (R)	0.01	−3.32***	0.03*
I find it difficult to work out people’s intentions	−0.11***	−12.99***	−0.07***

Pearson’s (*r*) correlations measure the association between each autistic trait and social psychological knowledge. Each coefficient is compared, using Pearson and Filon’s (*z*), to Gollwitzer et al.’s (1) correlation (*r* = 0.05) between overall trait autism and social psychological knowledge. Multiple linear regression [overall model: *F*(10, 6584) = 69.35, *R*^2^ = 0.095, *P* < 0.001] estimates the unique contribution (*β*) of each trait to social psychological knowledge while controlling for the other 9 traits. Variance inflation factors (all < 10) indicate that multicollinearity is not a concern, and the residuals are normally distributed in the regression. *P < 0.05, **P < 0.01, ***P < 0.001. “(R)” denotes reverse-scored items. Calculations use and are reported to 2 decimal places.

Our exploratory analysis therefore extends Gollwitzer et al.’s (1) study, shedding further light on autism-related processing of social information. It follows the authors’ suggestions that social knowledge may be acquired by nonsocial learning, and that some autistic traits and strengths (10) support processing of social information through compensatory mechanisms (3, 4). However, in contrast to Gollwitzer et al.’s central interpretation of their findings, we suggest that autism, as a unitary construct, is not a straightforward predictor of social psychological skills. Rather, we conclude that different autistic traits make divergent contributions to social psychological knowledge, reflecting the various strengths and difficulties linked with autism (5).
